# Environmental enrichment reduces adolescent anxiety- and depression-like behaviors of rats subjected to infant nerve injury

**DOI:** 10.1186/s12974-018-1301-7

**Published:** 2018-09-12

**Authors:** Xingrui Gong, Yongmei Chen, Jing Chang, Yue Huang, Meihau Cai, Mazhong Zhang

**Affiliations:** 1 Department of Anesthesiology and Pediatric Clinical Pharmacology Laboratory, Shanghai Children’s Medical Center, Shanghai Jiao Tong University School of Medicine, 1678 Dongfang Road, 200127 Shanghai, China; 2 Department of Anesthesiology, Shiyan Taihe Hospital (Affiliated Hospital of Hubei University of Medicine), Shiyan, Hubei China; 3 Institute of Anesthesiology, Department of Anesthesiology, Shiyan Taihe Hospital (Affiliated Hospital of Hubei University of Medicine), Shiyan, Hubei China; 4 Department of Laboratory, Shiyan Taihe Hospital (Affiliated Hospital of Hubei University of Medicine), Shiyan, Hubei China

**Keywords:** Environmental enrichment, Anxiety, Depression, Inflammation

## Abstract

**Background:**

Infant nerve injury causes delayed adolescent neuropathic pain, but whether it also leads to psychiatric illness is unknown. Environmental enrichment (EE) increases social communication and activity. Thus, our goal was to test anxiety- and depression-like behaviors after infant peripheral nerve injury and evaluate the effect of environmental enrichment on these models of affective disorders.

**Methods:**

Open field, elevated plus maze, sucrose preference, and pain behaviors (paw withdrawal threshold, spontaneous guarding score, and cold response to acetone) were measured in rats that received infant spared nerve injury (SNI). Enzyme-linked immune absorbent assay of cytokines was performed to evaluate the inflammatory response in the brain. Then, the ability of intracerebroventricular (ICV) injection of a microglia inhibitor, minocycline (MIN), and EE (a free-running wheel, a staircase, a plastic tunnel, a raised platform, and various colored balls) to reverse the infant SNI effects on behaviors and cytokines was examined.

**Results:**

Infant nerve injury resulted in adolescent anxiety- and depression-like behaviors. The medial prefrontal cortex, basolateral amygdala, and ventral hippocampus were skewed to a pro-inflammatory profile. ICV injection of MIN reduced anxiety- and depression-like behaviors without affecting pain behaviors. In addition, ICV MIN skewed the brain towards an anti-inflammatory profile. Finally, environmental enrichment improved anxiety- and depression-like behaviors, as well as pain behaviors. EE increased brain IL-10 and decreased IL-1β and TNF-α.

**Conclusions:**

Infant nerve injury induces adolescent anxiety- and depression-like behaviors and central nervous inflammation. Environmental enrichment reduces these behaviors by normalizing the inflammation balance in the brain.

## Background

Anxiety and depression are frequent comorbidities of chronic pain. About 40–60% of patients with chronic pain report anxiety and depression [1], and the prevalence of a lifetime history of psychiatric disorders is higher in chronic pain patients [2]. Both depression and chronic pain are debilitating and have a negative effect on quality of life [3], and both involve neuroinflammation. The causes of psychiatric disorders include monoamine dysfunction, hypothalamic-pituitary-adrenal axis dysfunction, deficiencies in neurogenesis, and neuroimmune activation [4]. Recently, these psychiatric disorders have been increasingly recognized as neuroinflammatory conditions because of the causes as described above and because they respond to anti-inflammatory treatment [5]. Microglia are the primary resident immune cells in the central nervous system. They are sentinels and patrol their surroundings constantly. After an insult, they change their morphology and release inflammatory cytokines [6, 7]. These inflammatory mediators affect neuron-neuron and neuron-glia communication and lead to affective disorders [5]. This opens up the possibility of treating these affective disorders by targeting microglia-related inflammation.

Neuroinflammation is also critical for the development of chronic pain including neuropathic pain conditions [8]. However, neuropathic pain is absent in infants. Peripheral nerve injury early in life often results in delayed onset of neuropathic pain behaviors starting from adolescence onward. For example, newborns with brachial plexus injury rarely develop chronic pain [9]; phantom pain happens long after congenital limb deficiency or early-life surgical amputation [10]. Population-based studies show that complex regional pain syndrome never occurs before adolescence [11]. Development of neuropathic pain is age-dependent, and chronic pain was rarely prevalent before adolescence [12]. In animal studies [13, 14], rats that received spared nerve injury (SNI) surgery did not develop neuropathic pain until 4 weeks after the injury, which was attributed to suppression of spinal inflammation in the early life. Considering that chronic pain is an important cause of psychiatric disorders [15] and that few clinical studies have focused on affective disorders induced by infant nerve injury, we evaluated anxiety- and depression-like behaviors after infant peripheral nerve injury in rats.

Anxiety and depression can have high morbidity and are often relapsing. Despite considerable effort dedicated to searching for efficacious treatment for these diseases, they are still refractory to treatment. Treating these conditions with psychiatric medications often brings an economic burden to families in addition to some severe side effects to patients. Environmental enrichment (EE) increases social communication and exploration [16], and a voluntary wheel running inhibits anxiety [17] and pain [18]. EE which includes a free-running wheel may be a useful approach to model this effect in preclinical studies. Thus, we tested the effect of EE on behaviors related to psychiatric disorders. Considering previous studies showing that anxiety and depression are inflammatory diseases [5, 6], we also measured the critical inflammatory cytokines IL-10, IL-1β, and TNF-α, after infant spared nerve injury (SNI), and measured changes in these cytokines in the central nervous system after EE treatment to explore potential mechanisms of EE effects.

## Methods

The study was approved by the institutional experimental animal use and care committee, Shanghai Children’s Medical Center, an affiliate of Shanghai Jiao Tong University School of Medicine, and was conducted under the guidelines of the National Institute of Health. All animals were kept in a controlled environment (25 °C, lights on from 8 AM to 8 PM) with free access to food and water.

**Fig. 1:**
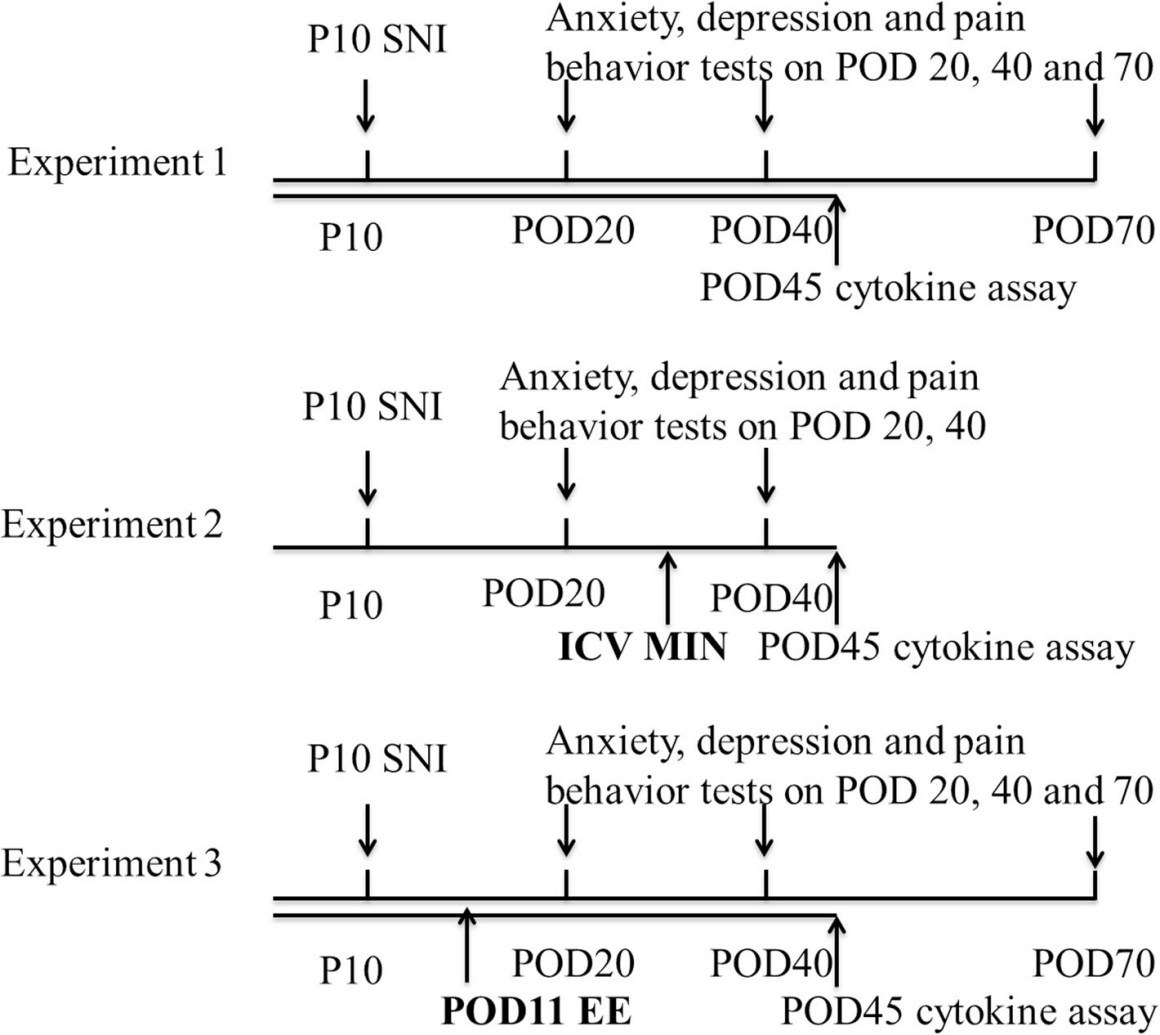
Experimental procedures and timeline. SNI surgery was performed by ligation and transection of the tibia and common peroneal branches, leaving the sural nerve intact. Experiment 1: Behavior tests and ELISA to measure the anxiety- and depression-like behaviors and inflammatory profile. Experiment 2: ICV MIN to evaluate the effect of a microglia inhibitor on the anxiety- and depression-like behaviors and inflammatory profile. Experiment 3: Test the effects of EE on anxiety- and depression-like behaviors and inflammatory profile of rats subjected to infant SNI. SNI spared nerve injury, ELISA enzyme-linked immune-absorbent assay, ICV intracerebroventricular, MIN minocycline, EE environmental enrichment, P postnatal days, POD postoperative days

Male Sprague-Dawley rat pups aged 1 week with their mothers and prior to weaning were purchased from the Shanghai Experimental Animal Center. The rat pups were numbered by their weight, then every two consecutive rats in weight order were randomly divided into sham or SNI groups by a computer-generated random number “1” or “2,” which indicated “sham” or “SNI” group, respectively. Each group was distributed across multiple litters (two to three animals) to allow for possible litter variability. Pups assigned into different groups remained with their single dam mothers until weaning. After weaning, only littermates were housed together in the same cage to avoid unnecessary stress. The surgery was performed as previously described [19]. On postnatal day (P) 10, the rat’s skin was prepared under isoflurane anesthesia on the right lateral thigh followed by a small incision to expose the sciatic nerve under a dissecting surgical microscope (Leica M205FA, Germany). The tibia and common peroneal branches were ligated and transected leaving the sural nerve intact. The skin was closed in layers and the rats were returned to their mother cages for recovery in the lab after the surgery, which typically lasted no more than 20–30 min. The cages were returned to the original animal facility on the same day. Rat pups were weaned at P21 and were housed two to three littermates per cage. Behavior tests, drug injections, and ELISA assays were done in a blinded fashion to avoid bias. The study consisted of three experiments as shown in Fig. 1.

In experiment 1, rat pups received sham or SNI surgery at P10, and in adolescence, rats received behavior tests on postoperative days (POD) 20, 40, and 70 (*n* = 8 rats in each group) or were sacrificed on POD 45 for cytokine assays of medial prefrontal cortex (mPFC), basolateral amygdala (BA), and ventral hippocampus (VH) (*n* = 6 rats in each group).

In experiment 2, rat pups received sham or SNI surgery at P10 and then received ICV injection of vehicle or minocycline (MIN) daily from POD 34 to POD 39; behavior tests were performed on POD 20 and POD 40. After the last behavior test on POD 45, all of the rats were decapitated and mPFC, BA, and VH were collected for cytokine assay (*n* = 10 rats per group).

In experiment 3, rat pups received sham or SNI surgery at P10, and EE (or conventional standard housing as a control) was employed starting from POD 11 to the end of the experiment. In four groups of animals, behavior tests were done on POD 20, 40, and 70 (*n* = 9 rats in each group). In another four groups of animals, rats were sacrificed for cytokine assays on POD 45 (*n* = 6 rats in each group). The EE consisted of a free-running wheel and any two of the following toys: a staircase, a plastic tunnel, a raised platform, and various colored balls. The two toys were changed every 3 to 5 days. The rats in the control groups were not exposed to the running wheel and toys.

### Pain behavior tests

Spontaneous guarding scores were measured as in a previous study [20]. Briefly, scores were 0 (paw flat on the floor with equal weight on both paws), 1 (mild shift of weight away from ipsilateral paw), 2 (unequal weight bearing and part of the paw lifted), or 3 (foot totally raised and not bearing any weight). Paw withdrawal threshold to mechanical stimulation was measured on POD 11,20, 40, and 70 in accordance with a previous study [18]. In the morning, the rats were placed in a Plexiglas box with a mesh floor 50 cm above the table. After 15 min of acclimation, the ipsilateral paw withdrawal thresholds were measured by an electric apparatus (IITC Life Science, USA). An increasing force was used to stimulate the ipsilateral hind paw, and when the paw withdrawal was observed, the value was recorded. Three repeated measures were averaged to get a mean value. Cold test response rate to acetone was done according to a previous study [20]; the score was recorded as withdrawal or licking or shaking of the hind paw to a drop of acetone applied to the ventral surface of the paw. The guarding behaviors were measured before the paw withdrawal threshold test and acetone test.

### Anxiety- and depression-like behavior tests

The open field test was performed on POD 20, 40, and 70 and followed the procedures described previously [21]. The rat was placed in a square arena (50 × 50 × 50 cm). The square was divided into two areas: a peripheral area and a central (17 × 17 cm) square area. The center distance and center duration were recorded as measures of anxiety-like behavior, and total running distance was recorded as a measure of general motor behavior. The running track was recorded for 5 min by a camera 2 m above the box. The field was cleaned with 75% ethanol after each test.

The elevated plus maze test was performed the day after the open field test to assess anxiety-like behavior as described previously [22]. The apparatus was comprised of two open arms and two closed arms (60 × 30 × 20 cm) emerging from a center platform (20 × 20 cm). The maze was covered with PVC material. After preparation, a rat was placed in the center of the platform area, and then, the track was videotaped for 5 min by a camera 1.5 m above the plus maze. The open arm entries and open arm duration were recorded for analysis. After each test, the rat was returned to its cage and the maze was cleaned with 75% ethanol. The videos of open field and elevated plus maze tests were analyzed by tracking software Noldus Ethovision XT (Noldus Information Technology, Leesburg, VA).

After the open field and elevated plus maze tests, the sucrose preference test was performed to assess depression-like behavior. The experiment followed a previous study with minor modifications [21]. Briefly, all of the rats were habituated with 1% sucrose for 48 h before experiments. On POD 25, 45, and 75, each rat received an 8-h fluid and food deprivation and then was exposed for 1 h to two bottles, one filled with 1%sucrose and the other tap water. The water and sucrose intakes were recorded to calculate the sucrose intake percentage (sucrose/(sucrose + water)) × 100%.

### Intracerebroventricular catheter placement and injection

Intracerebroventricular (ICV) catheter placement and injection followed the procedures used in a previous study [23]. Briefly, on POD 30, rats were anesthetized with pentobarbital sodium (50 mg/kg). After anesthesia, the rat was fixed on a stereotaxic apparatus. Then, the surgery was performed and a catheter was placed with stereotaxic coordinates: 0.8 mm posterior, 1.5 mm left lateral, and 4.5 mm ventral from the bregma. The guide cannula was fixed with dental cement and the rat was returned to its cage for recovery. The ICV cannulation was verified by injection of methylene blue through the cannula. The environmental enrichment toys were provided as before. Minocycline hydrochloride (purchased from Sigma-Aldrich Corp, St Louis, USA) was dissolved in normal saline. For each daily injection, rats were anesthetized with isoflurane as above; rats received bilateral microinjection of minocycline (160 μg/side) or saline (control) into the cerebral ventricle. A total volume of 4.0 μl was infused into each side over 10 min, and the injection syringe was left in place for an additional 5 min to allow for diffusion [23]. The microinjections were performed daily from POD34 to POD39. The behavior tests resumed on POD 40.

### Inflammatory mediators measurement by enzyme-linked immune-absorbent assay

On POD45, rats were deeply anesthetized with pentobarbital sodium (100 mg/kg) followed by decapitation. Tissues were collected for ELISA as previously described [24]. Briefly, the brains were placed in a chilled matrix and microdissected on a chilled glass plate. The mPFC was obtained from a 2-mm-thick slice ranging from approximately 5 to 3 mm anterior of Bregma. The VH was isolated from a 4-mm-thick section ranging from approximately 3.2 to 7.2 mm posterior of Bregma and was separated from cortex and underlying brain structures. The BA was dissected out ranging from approximately 1.2 to 3.2 mm posterior of Bregma just lateral to the optic tracks. After the samples were collected, these tissues were homogenized with normal saline and centrifuged at 2000 rpm, 4 °C for 10 min. The supernatants were used for IL-10, IL-1β, and TNF-α assay with ELISA kits (Nanjingjiancheng Biocompany, Nanjing, China) following the manufacturer’s instructions.

### Statistical analysis

The data are shown as mean ± SEM and were analyzed with one-way or two-way analysis of variance (ANOVA) with repeated measures. If a statistically significant difference was found, Bonferroni post hoc analysis was conducted. Nonparametric Wilcoxon or unpaired “*t*” test was used for comparison of variables without repeated measure. *P* < 0.05 was considered statistically significant. The statistical analysis was performed using GraphPad Prism software (GraphPad Prism 5.0, version 2.0; GraphPad Software Inc., San Diego, CA, USA).

## Results

### Infant SNI surgery induced adolescent anxiety- and depression-like behaviors

**Fig. 2:**
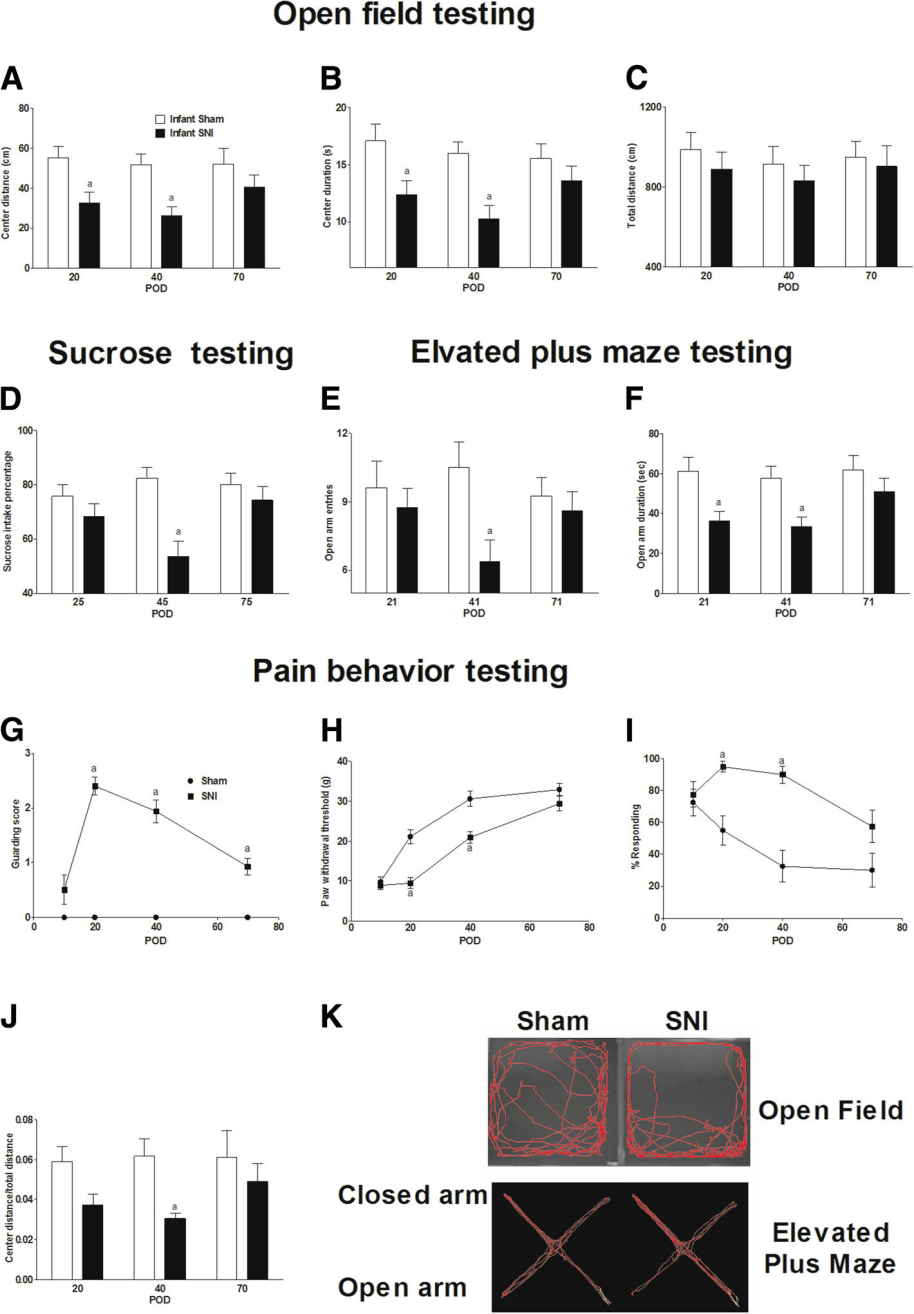
Infant SNI resulted in adolescent anxiety- and depression-like behaviors. Infant rats that received SNI on P10 showed decreased (**a**) center distance and (**b**) center duration compared to the sham group from POD 20 to POD 40. **c** The total distance traveled in the open field did not differ from the sham group. **d** Infant SNI surgery decreased sucrose intake percentage compared to the sham group on POD 45. In the elevated plus maze test, infant SNI surgery decreased (**f**) open arm duration compared to the sham group from POD 21 to POD 41 and (**e**) open arm entries on POD 41. In addition, infant SNI resulted in decreased (**g**) paw withdrawal threshold from POD 20 to POD 40, increased (**h**) guarding score from POD 20 to POD 70, and increased (**i**) cold sensitivity to acetone from POD 20 to POD 40. **j** SNI decreased the center distance/total distance calculated from the open field test on POD 40. Representative open field and elevated plus maze photos (**k**). *n* = 8. “a” denotes the statistically significant difference compared to the sham group

From adolescence onward, anxiety- and depression-like behaviors were measured. The open field test showed that infant SNI surgery decreased the center duration (group *F*_1,14_ = 13.2, *P* = 0.003; time *F*_2,28_ = 1.16, *P* = 0.329; interaction *F*_2,28_ = 1.29, *P* = 0.243) and center distance (group *F*_1,14_ = 11.4, *P* = 0.005; time *F*_2,28_ = 1.06, *P* = 0.359; interaction *F*_2,28_ = 1.07, *P* = 0.355) compared to the sham surgery group from POD 20 to POD 40 (Fig. 2a, b respectively). The total running distance in the open field was not different between the sham and SNI groups (Fig. 2c) (group *F*_1,14_ = 1.09, *P* = 0.315; time *F*_2,28_ = 0.326, *P* = 0.724; interaction *F*_2,28_ = 0.047, *P* = 0.955). SNI surgery decreased the center distance/total distance ratio (Fig. 2j) (group *F*_1,14_ = 7.7, *P* = 0.015; time *F*_2,28_ = 0.745, *P* = 0.484; interaction *F*_2,28_ = 0.737, *P* = 0.487), a measure which minimizes any small changes in total locomotion on the results. Representative open field and elevated plus maze photos are shown in Fig. 2k.

The elevated plus maze test showed that infant SNI surgery decreased open arm duration compared to the sham group from POD 21 to POD 41 (Fig. 2f) (group *F*_1,14_ = 8.38, *P* = 0.012; time *F*_2,28_ = 0.270, *P* = 0.766; interaction *F*_2,28_ = 1.763, *P* = 0.190). The number of open arm entries was decreased on POD 41 compared to the sham group (Fig. 2e) (group *F*_1,14_ = 11.6, *P* = 0.004; time *F*_2,28_ = 2.00, *P* = 0.154; interaction *F*_2,28_ = 1.08, *P* = 0.352). The sucrose preference test showed that the infant SNI surgery decreased the sucrose intake percentage on POD 45 compared to the sham group (Fig. 2d) (group *F*_1,14_ = 28.75, *P* = 0.000; time *F*_2,28_ = 1.55, *P* = 0.231; interaction *F*_2,28_ = 3.01, *P* = 0.653).

The paw withdrawal threshold (Fig. 2g) (group *F*_1,14_ = 42.6, *P* = 0.000; time *F*_3,42_ = 75.9, *P* = 0.000; interaction *F*_3,42_ = 4.97, *P* = 0.005) of rats subjected to infant SNI was decreased on POD 20 and POD 40, spontaneous guarding behavior (Fig. 2h) (group *F*_1,14_ = 404, *P* = 0.000; time *F*_3,42_ = 16, *P* = 0.000; interaction *F*_3,42_ = 16, *P* = 0.000) was increased on POD 20, POD 40, and POD 70, and the response to acetone (Fig. 2i) (group *F*_1,14_ = 53.8, *P* = 0.000; time *F*_3,42_ = 5.31, *P* = 0.003; interaction *F*_3,42_ = 2.94, *P* = 0.044) was increased on POD 20 and POD 40, as compared to sham rats.

### Infant SNI surgery increased mPFC, BA, and VH inflammatory mediators on POD 45

**Fig. 3:**
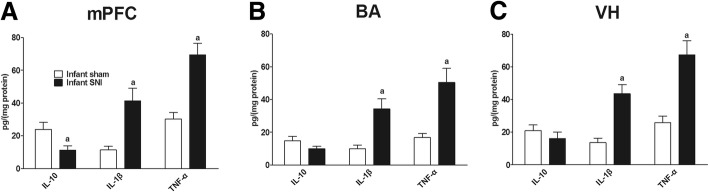
Infant SNI skewed mPFC, BA, and VH to a pro-inflammatory profile. On POD 45, infant SNI surgery increased **a** mPFC, **b** BA, and **c** VH IL-1β and TNF-α while decreasing mPFC and BA IL-10 expression compared to the sham group. *n* = 6. “a” denotes the statistically significant difference compared to the sham group

The ELISA results showed that SNI surgery increased IL-1β in the mPFC (*P* = 0.003, unpaired *t* test), BA (*P* = 0.004, unpaired *t* test), and VH (*P* = 0.001, unpaired *t* test) and TNF-α in the mPFC (*P* = 0.001, unpaired *t* test), BA (*P* = 0.003, unpaired *t* test), and VH (*P* = 0.001, unpaired *t* test) compared to the sham group (Fig. 3a–c). In contrast, infant SNI surgery decreased IL-10 in the mPFC compared to the sham group (*P* = 0.032, unpaired *t* test, Fig. 3a). These results showed that SNI caused the adolescent brain to skew towards a pro-inflammatory profile.

### ICV MIN reduced adolescent anxiety- and depression-like behaviors

**Fig. 4:**
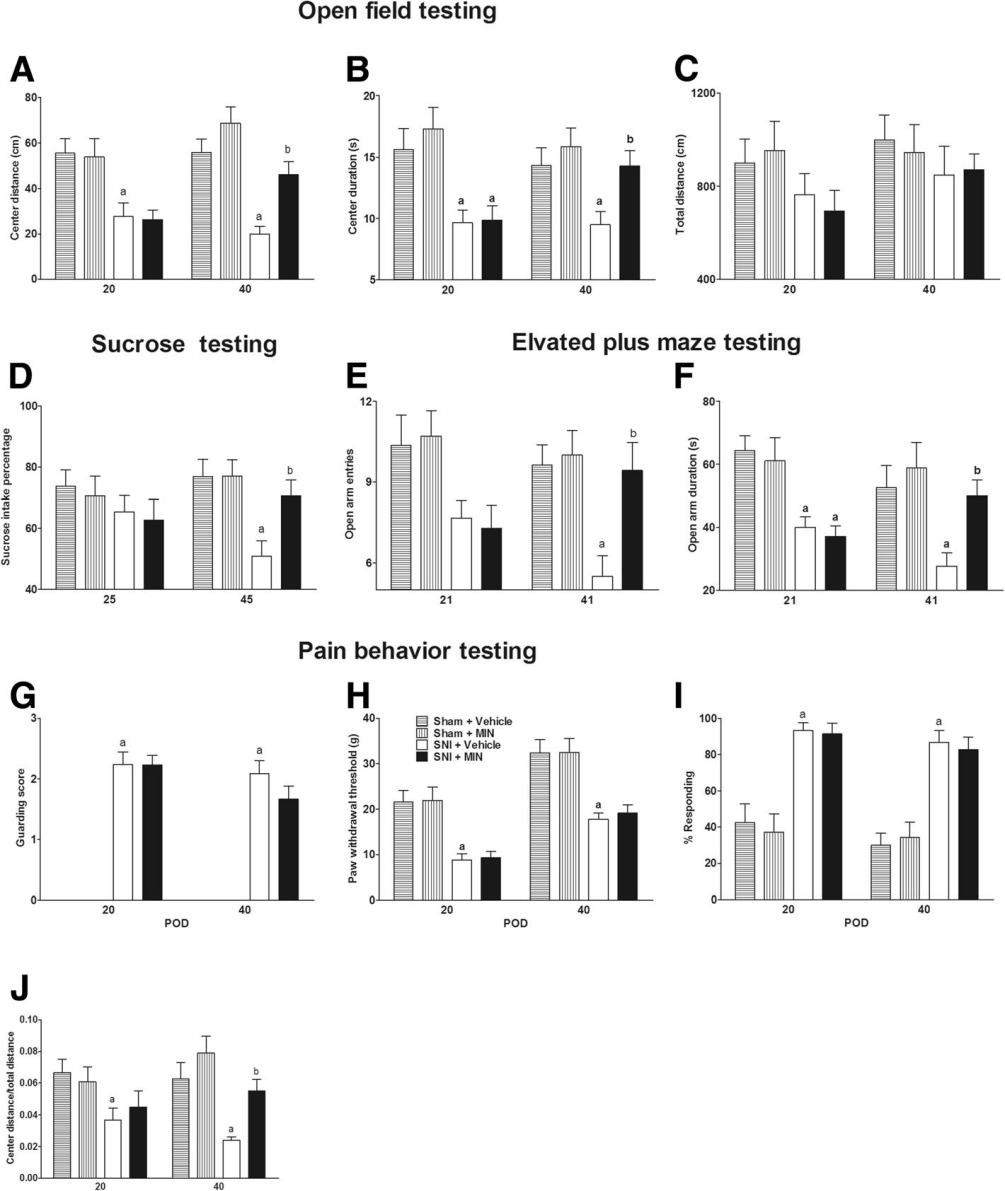
ICV MIN decreased infant SNI-induced anxiety- and depression-like behaviors. Infant SNI decreased **a** center duration and **b** distance from POD 20 to POD 40 in the open field test, **f** open arm duration from POD 20 to POD 40, **e** open arm entrances on POD 40 in the elevated plus maze test, and **d** sucrose intake percentage on POD 45. ICV MIN increased center duration and distance in the open field test, open duration and open entrance on POD 40 in the elevated plus maze test, and sucrose intake percentage on POD 45. ICV MIN did not affect the total distance traveled in the open field test (no difference between the four groups) and did not affect any behaviors of the sham rats. Infant SNI decreased **g** paw withdrawal threshold and **h** increased guarding score (guarding scores in the two sham groups were zero) and **i** response to acetone from POD 20 to POD 40, while ICV MIN did not affect these values in sham or SNI rats. **k** SNI decreased center/total distance compared to the sham group, while ICV MIN decreased that effect on POD40. *n* = 6–8. “a” denotes the statistically significant difference compared to the sham plus vehicle group. “b” denotes the statistically significant difference compared to the SNI plus vehicle group

In experiment 2, four rats did not complete the experiment and were excluded, including one in sham + MIN, one in SNI + MIN, and two in SNI + vehicle group. Two rats in the SNI + vehicle group died due to respiratory depression possibly caused by pentobarbital overdose. One rat in the sham + MIN group and one in the SNI + MIN group were excluded from the study due to failure of proper cannulation. The behavior results showed that ICV MIN for 6 days starting at POD34 did not affect anxiety, depression, and pain behaviors of sham rats (Fig. 4a–f). Consistent with experiment 1, SNI surgery induced adolescent anxiety, depression, and pain behaviors compared to sham rats. ICV MIN starting at POD 34 increased the center distance (group *F*_3,24_ = 15.3, *P* = 0.000; time *F*_1,24_ = 2.41, *P* = 0.133; interaction *F*_3,24_ = 2.00, *P* = 0.141) and center duration (group *F*_3,24_ = 8.42, *P* = 0.001; time *F*_1,24_ = 0.146, *P* = 0.706; interaction *F*_3,24_ = 1.867, *P* = 0.162) on POD 40 in the open field test, open arm duration (group *F*_3,24_ = 7.91, *P* = 0.000; time *F*_1,24_ = 0.28, *P* = 0.602; interaction *F*_3,24_ = 1.59, *P* = 0.216) and a number of open arm entries (group *F*_3,24_ = 7.1, *P* = 0.000; time *F*_1,24_ = 0.83, *P* = 0.37; interaction *F*_3,24_ = 2.54, *P* = 0.08) on POD 41 in the elevated plus maze test, and sucrose intake percentage (group *F*_3,24_ = 4.83, *P* = 0.001; time *F*_1,24_ = 0.02, *P* = 0.88; interaction *F*_3,24_ = 1.16, *P* = 0.35) on POD 45 in the sucrose preference test (Fig. 4a–f). Although SNI decreased the paw withdrawal threshold (group *F*_3,28_ = 17.93, *P* = 0.000; time *F*_1,28_ = 51.0, *P* = 0.000; interaction *F*_3,24_ = 0.105, *P* = 0.957) and increased spontaneous guarding behaviors (group *F*_3,28_ = 176.7, *P* = 0.000; time *F*_1,24_ = 3.52, *P* = 0.073; interaction *F*_3,24_ = 3.52, *P* = 0.140) and response to acetone (group *F*_3,28_ = 34.1, *P* = 0.000; time *F*_1,24_ = 1.63, *P* = 0.214; interaction *F*_3,24_ = 0.12, *P* = 0.947), ICV MIN did not affect those behaviors in the sham or SNI rats (Fig. 4g–i). SNI decreased the center/total distance (Fig. 4j) (group *F*_3,24_ = 5.93, *P* = 0.004; time *F*_1,24_ = 0.255, *P* = 0.618; interaction *F*_3,24_ = 1.49, *P* = 0.244) compared to the sham group, while ICV MIN decreased that effect on POD40.

### ICV MIN reduced mPFC, BA, and VH inflammation of rats subjected to infant SNI on POD 45 compared to ICV vehicle group

**Fig. 5:**
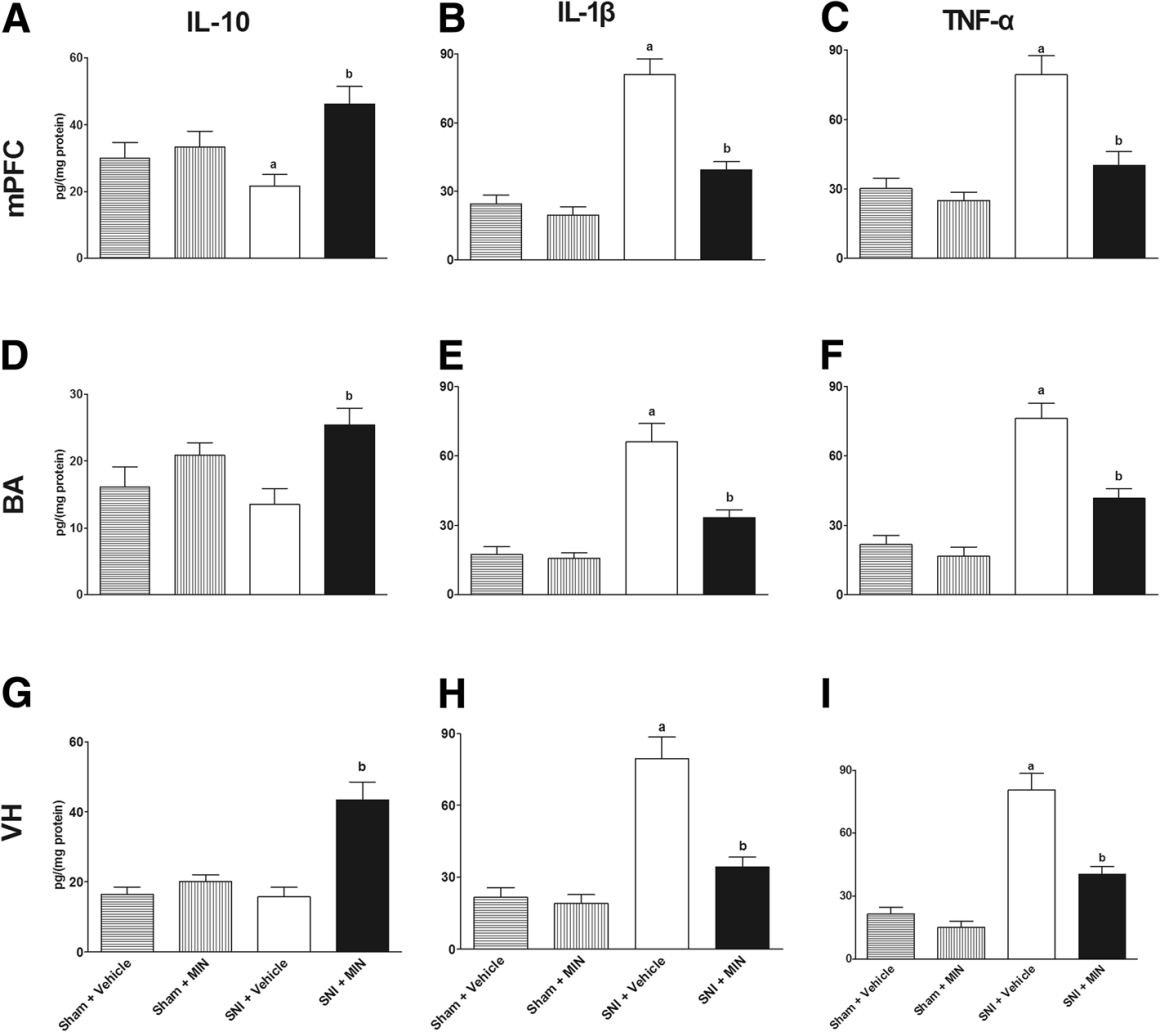
ICV MIN changed the mPFC, BA, and VH inflammatory profile. Infant SNI decreased IL-10 in mPFC, and ICV MIN increased IL-10 in the **a** mPFC, **d** BA, and **g** VH. Infant SNI increased IL-1β and TNF-α in mPFC, BA, and VH, and ICV MIN decreased IL-1β and TNF-α in the **b, c** mPFC, **e, f** BA, and **g, h** VH. *n* = 6–8. “a” denotes the statistically significant difference compared to the sham plus vehicle group. “b” denotes the statistically significant difference compared to the SNI plus vehicle group

After the behavior tests on POD 45, rats were decapitated and samples were collected for ELISA tests. The ELISA results showed infant SNI increased IL-1β and TNF-α in mPFC (Fig. 5a–c), BA (Fig. 5d–f), and VH (Fig. 5g–i), while ICV MIN increased IL-10 in mPFC (*F*_3,24_ = 4.47, *P* = 0.013), BA (*F*_3,24_ = 4.14, *P* = 0.017), and VH (*F*_3,24_ = 16.23, *P* = 0.000) and decreased IL-1β in mPFC (*F*_3,24_ = 34.85, *P* = 0.000), BA (*F*_3,24_ = 24.87, *P* = 0.000), and VH (*F*_3,24_ = 25.52, *P* = 0.000) and TNF-α in mPFC (*F*_3,24_ = 17.41, *P* = 0.000), BA (*F*_3,24_ = 31.2, *P* = 0.000), and VH (*F*_3,24_ = 39.6, *P* = 0.000). The increased IL-10 and decreased IL-1β and TNF-α in mPFC, BA, and VH suggested that ICV MIN skewed the SNI-induced pro-inflammatory profile towards an anti-inflammatory profile in those regions.

### EE inhibited infant SNI-induced adolescent pain and anxiety- and depression-like behaviors

**Fig. 6:**
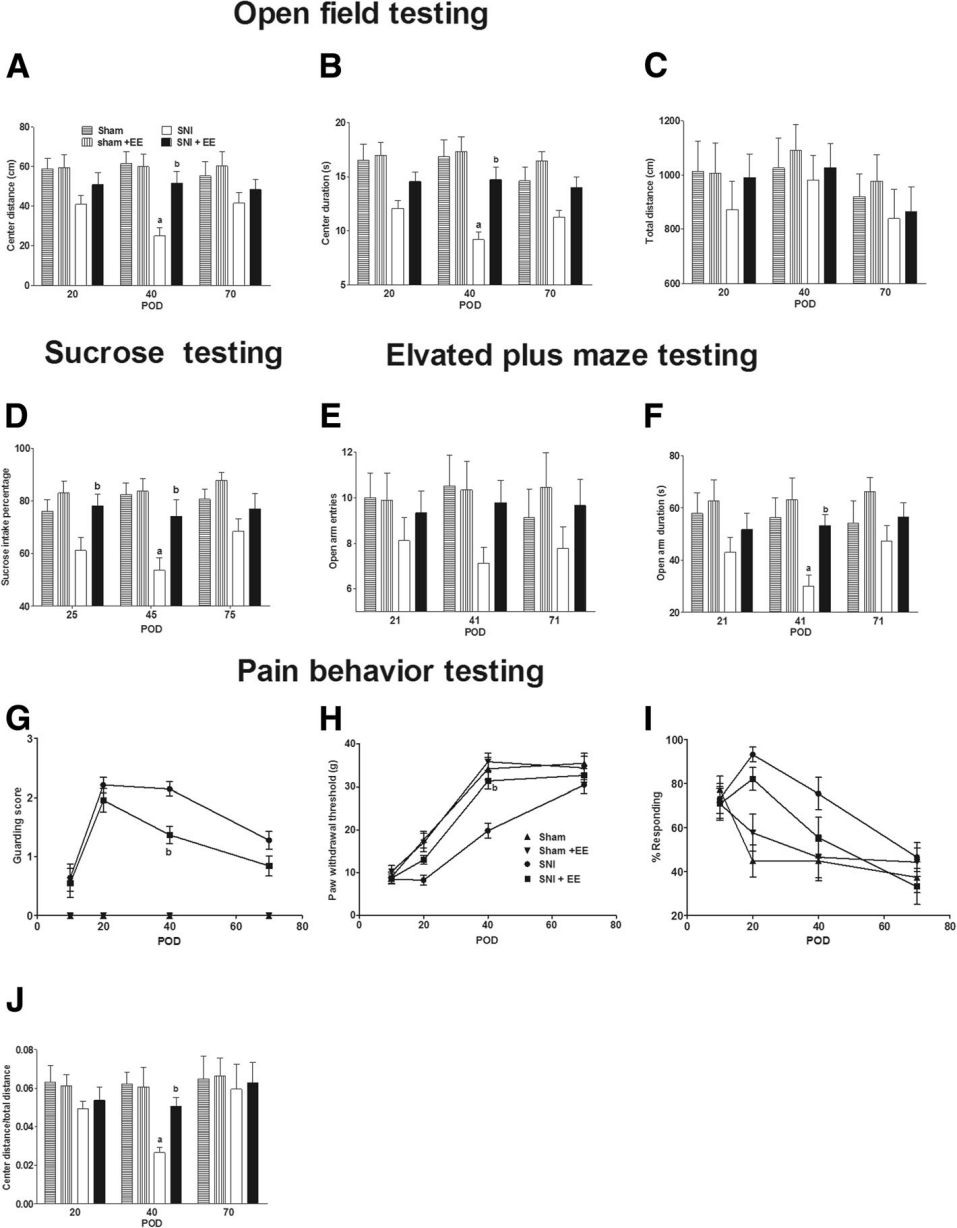
Environmental enrichment (EE) reduced infant SNI-induced anxiety- and depression-like behavior. After EE intervention starting from POD11, **a** the center distance and **b** duration were greatly increased on POD 40 in the open field test compared to the group without EE. **c** The total distance was not different between the four groups. **d** EE increased sucrose intake percentage compared to the group without EE from POD 25 to POD 45. **f** Open arm duration was increased on POD 41 after EE when compared to the group without EE. After EE, **h** the guarding score was decreased and **g** the paw withdrawal threshold was increased on POD 40 as compared to the SNI group. **i** EE did not decrease the response to acetone of rats subjected to infant SNI. **k** SNI decreased center/total distance compared to the sham group, while ICV MIN decreased that effect on POD40. *n* = 8–9. “a” and “b” denote the statistically significant difference compared to the sham and SNI group, respectively

In the EE intervention experiment, one rat in the sham group was excluded because of incision infection. After EE starting from POD 11, the center duration (group *F*_3,31_ = 16.2, *P* = 0.000; time *F*_2,62_ = 0.285, *P* = 0.824; interaction *F*_6,62_ = 0.913, *P* = 0.491) and center distance (group *F*_3,31_ = 9.67, *P* = 0.000; time *F*_2,62_ = 0.285, *P* = 0.753; interaction *F*_6,62_ = 0.454, *P* = 0.968) on POD 40 were greatly increased compared to the group without EE in the open field test of rats that received SNI on POD 10 (Fig. 6a, b). The total running distance in the open field was not different between the four groups (Fig. 6c) (group *F*_3,31_ = 0.593, *P* = 0.624; time *F*_2,62_ = 2.38, *P* = 0.101; interaction *F*_6,62_ = 0.111, *P* = 0.995). EE increased open arm duration (group *F*_3,31_ = 5.79, *P* = 0.003; time *F*_2,62_ = 0.744, *P* = 0.480; interaction *F*_6,62_ = 0.566, *P* = 0.756) compared to the group without EE on POD 41 in the elevated plus maze test of rats that received SNI on P10 (Fig. 6f). The sucrose preference test showed that EE increased sucrose intake percentage on POD 25 and POD 45, compared to the group without EE of rats that received SNI on P10 (Fig. 6d) (group *F*_3,31_ = 12.49, *P* = 0.000; time *F*_2,62_ = 1.305, *P* = 0.278; interaction *F*_6,62_ = 0.762, *P* = 0.603). EE did not affect behavior results of sham rats in the open field, elevated plus maze, and sucrose preference tests. SNI decreased center/total distance compared to the sham group, while EE decreased that effect on POD40 in the open field test (Fig. 6k) (group *F*_3,31_ = 3.33, *P* = 0.042; time *F*_2,62_ = 2.12, *P* = 0.118; interaction *F*_6,62_ = 0.967, *P* = 0.455).

In addition, EE increased the paw withdrawal threshold (Fig. 6g) (group *F*_3,31_ = 12.49, *P* = 0.000; time *F*_3,93_ = 185.4, *P* = 0.000; interaction *F*_9,93_ = 2.97, *P* = 0.004) and decreased the spontaneous guarding score (Fig. 6h) (group *F*_3,31_ = 160.1, *P* = 0.000; time *F*_3,93_ = 26.32, *P* = 0.000; interaction *F*_9,93_ = 9.67, *P* = 0.000) on POD 40 compared to the group without EE of rats that received infant SNI. Although SNI increased the response to acetone (Fig. 6i) (group *F*_3,31_ = 6.81, *P* = 0.001; time *F*_3,93_ = 16.1, *P* = 0.000; interaction *F*_9,93_ = 0.019, *P* = 2.36), EE did not affect the response to acetone of sham rats or rats that received SNI.

### EE skewed mPFC, BA, and VH to an anti-inflammatory profile on POD 45

**Fig. 7:**
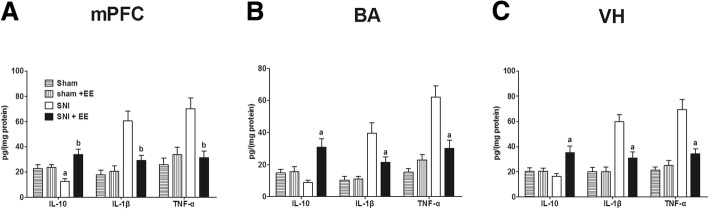
EE skewed the brain to an anti-inflammatory profile. On POD 45, EE increased IL-10 expression and decreased IL-1β and TNF-α expression in **a** mPFC, **b** BA, and **c** VH compared to without EE. *n* = 6. “a” and “b” denote the statistically significant difference compared to the sham and SNI group, respectively

After EE intervention from POD21 to POD45, the rats were killed and decapitated on POD45, and samples were obtained from three brain regions and prepared for ELISA assays. The ELISA results showed that EE increased IL-10 in mPFC (*F*_3,20_ = 8.80, *P* = 0.001), BA (*F*_3,20_ = 7.99, *P* = 0.001), and VH (*F*_3,20_ = 5.78, *P* = 0.001) and decreased IL-1β in mPFC (*F*_3,20_ = 14.5, *P* = 0.000) and VH (*F*_3,20_ = 17.1, *P* = 0.000) and TNF-α in mPFC (*F*_3,20_ = 9.85, *P* = 0.001), BA (*F*_3,20_ = 18.2, *P* = 0.000), and VH (*F*_3,20_ = 19.0, *P* = 0.000) of rats that received infant SNI (Fig. 7a–c). EE did not affect IL-10, IL-1β, and TNF-α in the mPFC, BA, and VH of sham rats.

## Discussion

Our study showed that the infant nerve injury resulted in anxiety- and depression-like behaviors in adolescence, while EE reduced adolescent anxiety- and depression-like behaviors and suppressed central nervous inflammation.

In the study, we found that infant rats with peripheral nerve injury exhibited anxiety- and depression-like behaviors. Anxiety-like behaviors, including open field test and elevated plus maze test measures, peaked on POD 40 and disappeared on POD 70. Sucrose intake percentage, a measure of depression-like behavior, was decreased on POD 45. The total distance traveled was not different between groups in the open field test, which excluded the possibility that anxiety- and depression-like results were caused by motor impairment. In addition, IL-10 decreased in mPFC and BA, while IL-1β and TNF-α protein expression increased in mPFC, BA, and VH on POD 45, which indicated a CNS shift to pro-inflammatory profile at the time when anxiety- and depression-related behaviors were observed. In addition, our results showed neuropathic pain generally coincided with these behaviors. Since neuropathic pain causes psychiatric disorders [25], it is reasonable to deduce, at least in part, that the delayed neuropathic pain contributed to the anxiety- and depression-like behaviors.

Previous studies have demonstrated substantive links between neuroinflammation and psychological disorders [26]. Microglia are a major source of inflammation in the central nervous system [27], and the application of MIN, a microglia inhibitor, abolished anxiety- and depression-like behavior in a previous study [28]. Thus, we tested injection of MIN to treat anxiety- and depression-like behaviors in rats subjected to infant peripheral nerve injury. Our results showed that ICV MIN reduced adolescent anxiety- and depression-like behaviors induced by infant SNI surgery. This is in agreement with some previous clinical and preclinical studies showing anxiety and depression are associated with microglia activation and are reduced by MIN [6]. In addition, we found that ICV MIN reduced mPFC, BA, and VH inflammation. The inflammatory profile switch was verified by our ELISA results showing that MIN decreased IL-1β and TNF-α protein expression and increased IL-10 expression. These effects on the cytokine profile are similar to those found in a preclinical study of systemic MIN that showed it shifted brain microglia towards an M2 phenotype [29]. However, a limitation of our study is that we did not directly examine brain microglia and cannot exclude other mechanisms such as direct effects on neurons [30]. In contrast, our results showed that ICV MIN did not affect pain behaviors; this may be because the effects of the small dose of MIN were limited to microglial inhibition in the brain only, rather than influencing microglia in the spinal cord, so that MIN did not affect lower extremity reflexes. Another reason may be that MIN is effective for the prevention of neuropathic pain development, but has no effect on established neuropathic pain [31]. Our results showed ICV MIN did not affect delayed adolescent neuropathic pain behaviors induced by infant nerve injury when applied after these behaviors were already established.

After infant nerve injury, EE was employed to counteract the anxiety- and depression-related behaviors. Our results showed that EE is anxiolytic and anti-depressive. On POD 40, the anxiety- and depression-like behaviors were improved. However, on POD 20, the anxiety-like behaviors were not different between groups, possibly because this time point was only 9 days after the start of EE. The ELISA results on POD45 support the behavioral findings and showed decreased proinflammatory cytokines after EE. Previous studies have demonstrated that anxiety and depression are inflammatory diseases and that modulation of inflammation is anxiolytic and anti-depressive [5]. IL-1β and TNF-α are pro-inflammatory cytokines. ICV application of these cytokines provokes anxiety- and depression-like behaviors [32, 33], whereas the ICV application of IL-10, an anti-inflammatory cytokine, abolished these behaviors [34]. This is consistent with our results showing that EE decreased both the anxiety- and depression-like behaviors and inflammation in the brain. Our results indicate EE is anxiolytic and antidepressant and it promotes a switch from a pro-inflammatory to an anti-inflammatory profile in the brain.

Previous studies have demonstrated IL-1β is an important pro-inflammatory cytokine in the formation of psychiatric disorders: blocking IL-1β signaling with an antagonist greatly reduced inflammatory psychiatric disorders, and drugs targeting IL-1β are in phase II clinical trials [33, 35]. TNF-α is another pro-inflammatory cytokine that plays a pivotal role in the development of psychiatric disorders. A single dose of TNF-α by ICV injection induced anxiety- and depression-like behaviors, while antagonizing TNF-α abolished those behaviors [32, 36]. In addition, TNF-α is the main cytokine involved in the genesis of chronic pain. Blocking TNF-α not only reduced psychiatric disorders but also reduced chronic pain at the same time [15]. In contrast, IL-10 is an anti-inflammatory cytokine and is decreased in various affective disorders, whereas central administration of IL-10 antagonized these disorders [34], an effect attributed to the suppression of neuroinflammation. Consistently, IL-10^−^/^−^ animals displayed increased depressive-like behavior that was reversed by IL-10. Moreover, mice overexpressing IL-10 showed a decreased depressive-like behavior [37]. Our results showed that EE and ICV MIN switched the brain from a pro-inflammatory towards an anti-inflammatory profile, reversing the increases in IL-1β and TNF-α and decreases in IL-10. Generally, the results confirmed that affective disorders are inflammatory illnesses, and modulation of inflammation is effective for the treatment of affective illnesses [5, 38].

Our results showed that EE also increased the adolescent pain threshold of rats subjected to infant nerve injury. Previous studies demonstrated that environmental enrichment [39] and exercise [40] both relieve neuropathic pain. Thus, the increased pain threshold we observed could be due to the environment and/or voluntary running. Exercise has been known to have mild to moderate anxiolytic and antidepressant effect. Possible mechanisms include changes in monoamine levels, altered levels of stress hormones (e.g., cortisol), and upregulation of neurotrophic factors [41]. A recent meta-analysis study reported that exercise is effective in treating depression in adolescents [42]. Of course, larger trials with clinical samples that adequately minimize the risk of bias are needed to confirm the findings. Exercise is also known to have neuroprotective effects by increasing expression of anti-inflammatory cytokines and decreasing levels of pro-inflammatory cytokines [43]. Since anxiety and depression are increasingly being recognized as inflammatory conditions [26], it is reasonable to believe that exercise-induced inhibition of inflammatory responses contributes to the anxiolytic and antidepressant effects observed in the current study. Since EE is also anti-inflammatory [44] and EE in our protocol includes exercise [22], our study does not distinguish which was the most important in alleviating anxiety and depression behaviors.

Our results showed that infant nerve injury induced adolescent anxiety- and depression-like behaviors. We tested these behaviors beginning at POD20, to avoid possible stress caused by separation from their mothers. In addition, the anxiety- and depression-like behaviors were not permanent and disappeared on POD 70 in untreated rats. This may be due to the following reasons. Chronic pain is an important contributor to anxiety and depression, and with age, chronic pain decreases [14]. Consequently, the rat anxiety- and depression-like behaviors may also improve with age. One of the limitations of the study is that we do not know whether the anxiolytic and anti-depressive effect of EE is due to suppression of brain inflammation, reduced neuropathic pain, or a combination of the two. The relationship between chronic pain and psychiatric illness is rather complex. Chronic pain leads to psychiatric illness, whereas psychiatric illness sensitizes chronic pain. These two disorders often exacerbate each other, leading to a vicious cycle that is refractory to medical treatment [15]. Although it is well recognized that chronic pain and psychiatric illness co-exist, it is not fully explored as to why or how they do so. A better understanding of their relationship may help to resolve both problems. However, our results showed EE reduces adolescent anxiety- and depression-like and neuropathic pain behaviors of rats subject to infant nerve injury.

We targeted mPFC, BA, and VH because many studies have demonstrated that these regions are key sites of inflammation in psychiatric conditions, while suppression of pro-inflammatory cytokines in those sites decreases various affective disorders [15, 45]. mPFC and BA are undergoing continuing development during adolescence [46]. This late development may lead to susceptibilities to psychopathologies that often develop at this time. A previous study [47] has demonstrated that IL-1β is increased in mPFC in chronic stress-treated rats, while decreasing IL-1β in mPFC also decreased anxiety- and depression-like behaviors. Similarly, others report that mice with complete Freund’s adjuvant-induced inflammatory pain showed anxiety-like behavior and increased TNF-α in the BA [48]. VH has been reported to participate in psychiatric disorders by sending information to mPFC and BA [49]. Lesions of the ventral hippocampus but not the dorsal hippocampus attenuate anxiety behaviors and prevent the physiological response of increased defecation in response to an anxiogenic environment [50, 51]. Therefore, in our study, those regions were chosen to evaluate the inflammatory conditions.

To avoid estrus cycle effects, only male rats were included in our study. However, recent studies from other groups showed that pain and psychiatric disorders exhibit sex differences. Microglia play a key role in chronic pain initiation and maintenance in males, while in females, adaptive immune cells, likely T lymphocytes, exert a more important role in chronic pain. Estrogen and progesterone suppress innate immunity, and testosterone inhibits adaptive immunity which may contribute to these effects [52]. Similar to pain, anxiety and depression also showed sex differences. Previous studies have demonstrated that females do not show anxiety-like behaviors and had less inflammation [53]. This was attributed to the suppression of neuroinflammation by estradiol [54]. Moreover, large-scale data from the Netherlands Study of Depression and Anxiety reported that inflammation associated with depression is male-specific [55]. Despite sexual dimorphisms in the prevalence of psychiatric illnesses, much current research investigating the neuroimmune impact of stress remains exclusively focused on male subjects. However, in our results, we found that male rat brains skewed to a proinflammatory profile when they presented anxiety- and depression-like behaviors.

## Conclusions

In summary, results from our study demonstrate that infant nerve injury induces adolescent anxiety- and depression-like behaviors and that the CNS switches to a proinflammatory profile. Suppression of microglia-related inflammation by MIN counteracts the adolescent anxiety- and depression-like behaviors. EE including exercise, a non-pharmacological method, inhibits adolescent anxiety- and depression-like behaviors and restores the balance of inflammatory mediators.

## Abbreviations

ANOVA: Analysis of variance
BA: Basolateral amygdala
EE: Environmental enrichment
ELISA: Enzyme-linked immune-absorbent assay
ICV: Intracerebroventricular
MIN: Minocycline
mPFC: Medial prefrontal cortex
P: Postnatal day
POD: Postoperative day
SNI: Spared nerve injury
VH: Ventral hippocampus
